# Supplementary material for: Endoplasmic reticulum–plasma membrane contact gradients direct cell migration
Source: Nature. 2024 Jun 12;631(8020):415–23. doi: 10.1038/s41586-024-07527-5 (PMC11236710; doi:10.1038/s41586-024-07527-5)

nature portfolio

https://doi.org/10.1038/s41586-024-07527-5

## Supplementary information

# Endoplasmic reticulum–plasma membrane contact gradients direct cell migration

In the format provided by the authors and unedited

**a** Extended Data Fig.3d

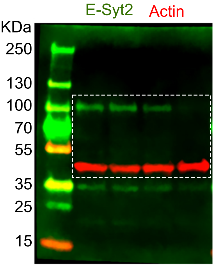


**b** Extended Data Fig.4a

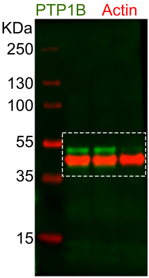


**c** Extended Data Fig.7a

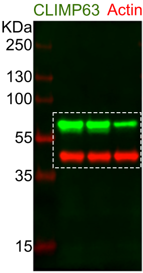


**d** Extended Data Fig.7b

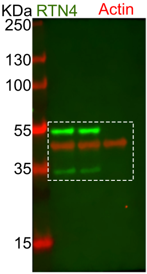

Supplement: Supplementary file 1 — Uncropped source western blot images. Combined source images for figures in Extended Data Fig. 3d (source data: Supplementary Fig. 1a), 4a (source data: Supplementary Fig. 1b), 7a (source data: Supplementary Fig. 1c) and 7b (source data: Supplementary Fig. 1d). Primary antibodies were used as indicated and detected in the same gel. [file 41586_2024_7527_MOESM1_ESM.pdf]
