# Supplementary material for: Endoplasmic reticulum–plasma membrane contact gradients direct cell migration
Source: Nature. 2024 Jun 12;631(8020):415–23. doi: 10.1038/s41586-024-07527-5 (PMC11236710; doi:10.1038/s41586-024-07527-5)
Supplement: Supplementary file 2 — Reporting Summary [file 41586_2024_7527_MOESM2_ESM.pdf]

nature portfolio

Corresponding author(s): Tobias Meyer & Bo Gong

Last updated by author(s): Apr 14, 2024

Reporting Summary

Nature Portfolio wishes to improve the reproducibility of the work that we publish. This form provides structure for consistency and transparency in reporting. For further information on Nature Portfolio policies, see our Editorial Policies and the Editorial Policy Checklist.

Statistics

For all statistical analyses, confirm that the following items are present in the figure legend, table legend, main text, or Methods section.

n/a	Confirmed	
☐	☒	The exact sample size (*n*) for each experimental group/condition, given as a discrete number and unit of measurement
☐	☒	A statement on whether measurements were taken from distinct samples or whether the same sample was measured repeatedly
☐	☒	The statistical test(s) used AND whether they are one- or two-sided<br>*Only common tests should be described solely by name; describe more complex techniques in the Methods section.*
☐	☒	A description of all covariates tested
☒	☐	A description of any assumptions or corrections, such as tests of normality and adjustment for multiple comparisons
☐	☒	A full description of the statistical parameters including central tendency (e.g. means) or other basic estimates (e.g. regression coefficient) AND variation (e.g. standard deviation) or associated estimates of uncertainty (e.g. confidence intervals)
☐	☒	For null hypothesis testing, the test statistic (e.g. *F*, *t*, *r*) with confidence intervals, effect sizes, degrees of freedom and *P* value noted<br>*Give P values as exact values whenever suitable.*
☒	☐	For Bayesian analysis, information on the choice of priors and Markov chain Monte Carlo settings
☒	☐	For hierarchical and complex designs, identification of the appropriate level for tests and full reporting of outcomes
☐	☒	Estimates of effect sizes (e.g. Cohen's *d*, Pearson's *r*), indicating how they were calculated

Our web collection on statistics for biologists contains articles on many of the points above.

Software and code

Policy information about availability of computer code

Data collection	Data shown in Fig. 2g, 2h, 3e, 3f, Extended Data Fig. 3c, f-i and Extended Data Fig.7 d,e were captured by the NIS-Elements AR 5.21.03 controlled by a Ti2-E inverted microscope (Nikon) equipped with an LED light source (Lumencor Spectra X) and Hamamatsu ORCA-Flash4.0 V3 sCMOS camera. Data shown in Extended Data Fig.2 a,b were captured on a TIRF microscope (Nikon) equipped with 488/561/647 nm lasers and high numerical aperture objective in the Rockefeller University's Bio-Imaging Resource Center. Other imaging experiments were performed on a SoRa spinning-disk confocal microscope (Marianas system, 3i), equipped with a Zeiss Axio Observer 7 stand, ORCA-Fusion BT sCMOS Camera (Hamamatsu), CSU-W1 SoRa confocal scanner unit (Yokogawa), 405/445/488/514/561/637 nm LaserStack (3i) and an extra 'Vector' device for photomanipulation operated by the Slidebook 6 software. Cryo-patterned grids were visualized with a Titan Krios electron microscope (Thermo Fisher scientific) equipped with a field emission gun, a GIF Quantum LS postcolumn energy filter (Gatan), and a K3 summit electron detector (Gatan). The electron microscope was operated at 300 kv in nanoprobe mode at a magnification of 19,500x (pixel size of 4.53 Å at the specimen level).
Data analysis	Automated analysis of time-lapse imaging was performed using a custom MATLAB R2021 pipeline, reported in Bisaria, A., et al., Science 368, 1205-1210, (2020) and Chung et al., Mol Cell 76, 562-573,(2019) by our group. For significance test and non-linear regression analysis, data were processed by GraphPad Prism 9 or MATLAB R2021. The raw Cryo-ET tilt movies were motion and CTF-corrected in Warp 1.09, aligned with the AreTomo software package and further reconstructed with either weighted-back projection (WBP) in AreTomo or with simultaneous iterative reconstructive technique (SIRT) using Tomo3D.

For manuscripts utilizing custom algorithms or software that are central to the research but not yet described in published literature, software must be made available to editors and reviewers. We strongly encourage code deposition in a community repository (e.g. GitHub). See the Nature Portfolio guidelines for submitting code & software for further information.

Data

Policy information about availability of data

All manuscripts must include a data availability statement. This statement should provide the following information, where applicable:

- Accession codes, unique identifiers, or web links for publicly available datasets
- A description of any restrictions on data availability
- For clinical datasets or third party data, please ensure that the statement adheres to our policy

The authors declare that all data supporting the findings of this study and source data in the excel file are available within the article and its supporting information files (extended data and supplementary videos).

Research involving human participants, their data, or biological material

Policy information about studies with human participants or human data. See also policy information about sex, gender (identity/presentation), and sexual orientation and race, ethnicity and racism.

Reporting on sex and gender	n/a
Reporting on race, ethnicity, or other socially relevant groupings	n/a
Population characteristics	n/a
Recruitment	n/a
Ethics oversight	n/a

Note that full information on the approval of the study protocol must also be provided in the manuscript.

Field-specific reporting

Please select the one below that is the best fit for your research. If you are not sure, read the appropriate sections before making your selection.

☒ Life sciences ☐ Behavioural & social sciences ☐ Ecological, evolutionary & environmental sciences

For a reference copy of the document with all sections, see nature.com/documents/nr-reporting-summary-flat.pdf

Life sciences study design

All studies must disclose on these points even when the disclosure is negative.

Sample size	Sample size for each experiment is indicated in figure legend. No statistical method was used to predetermine sample size and sample sizes were chosen based on pilot experiments and literature reports, sufficient for statistical analysis in our cell biology study.
Data exclusions	No data were excluded from our analysis.
Replication	All experiments were performed at least 3 times, but some data shown in the figures are one representative data from a single assay as indicated in the legend.<br>The data presented were successful in all 3 attempts or in the case of one failure, it was performed a fourth time to confirm the results to support conclusions stated in the manuscript.
Randomization	Randomization was not relevant for this study; Each experiment compared data sets from control conditions versus a perturbation(KD, inhibitor or over-experssion). Cells were selected based on their treatments.
Blinding	Investigators were not blinded for data collection, since the investigators were aware of sample and treatment allocations while setting up the experiments.

Reporting for specific materials, systems and methods

We require information from authors about some types of materials, experimental systems and methods used in many studies. Here, indicate whether each material, system or method listed is relevant to your study. If you are not sure if a list item applies to your research, read the appropriate section before selecting a response.

Materials & experimental systems

n/a	Involved in the study	
☐	☒	Antibodies
☐	☒	Eukaryotic cell lines
☒	☐	Palaeontology and archaeology
☒	☐	Animals and other organisms
☒	☐	Clinical data
☒	☐	Dual use research of concern
☒	☐	Plants

Methods

n/a	Involved in the study	
☒	☐	ChIP-seq
☒	☐	Flow cytometry
☒	☐	MRI-based neuroimaging

Antibodies

Antibodies used	Phospho-Tyrosine (P-Tyr-1000) MultiMab Rabbit mAb Mix (1:500, Cell Signaling Technology, #8954) and CLIMP63 monoclonal antibody (G1/296) (1:500, Enzo Life Sciences, ENZ-ABS669-0100). Anti-ESYT1 antibody (1:200, Sigma, HPA076926), Anti-ESYT2 antibody (1:1000, Sigma, HPA002132), PTP1B antibody (1:1000, BD Bioscience, 610139), RTN4 antibody (1:1000, Thermo Scientific, # MA5-32763). Secondary antibodies included goat anti-rabbit IgG(H+L) Alexa Fluor 568, Invitrogen (1:2000, Thermo Scientific, A-11011), goat anti-rabbit IgG(H+L) Alexa Fluor 647, Invitrogen (1:2000, Thermo Scientific, A-21245) and goat anti-rabbit IgG(H+L) Alexa Fluor 700, Invitrogen (1:2000, Thermo Scientific, A-21038).
Validation	All antibodies are commercially available and validated by the authors using western blot or immunofluorescence assay shown in the paper. All antibodies were used in multiple experiments to detect the indicated target protein giving results consistent to the expected moelcular weight, subcellular localization or signal changes after inhibitor treatment or siRNA knockdown.

Eukaryotic cell lines

Policy information about cell lines and Sex and Gender in Research

Cell line source(s)	hTERT RPE-1 cells (here referred to as RPE-1) (ATCC CRL-4000), HUVEC/TERT2 cells (ATCC CRL-4053), BJ-5ta cells (ATCC CRL-4001) and 293T cells (Takara Bio 632180) were used in this study.
Authentication	All cell lines were received directly from the supplier and other stable cell lines were constructed based on the original cell lines.
Mycoplasma contamination	All cell lines were verified to be mycoplasma free.
Commonly misidentified lines (See ICLAC register)	No commonly misidentified cell lines were used.
